# Dissociation and Suicidality in Eating Disorders: The Mediating Function of Body Image Disturbances, and the Moderating Role of Depression and Anxiety

**DOI:** 10.3390/jcm10174027

**Published:** 2021-09-06

**Authors:** Yael Doreen Lewis, Shirley Kapon, Adi Enoch-Levy, Amit Yaroslavsky, Eliezer Witztum, Daniel Stein

**Affiliations:** 1 Hadarim Eating Disorders Center, Shalvata Mental Health Center, Hod Hasharon 4534708, Israel; yael.d.lewis@gmail.com; 2 Department of Psychiatry, Sackler Faculty of Medicine, Tel Aviv University, Tel Aviv 69978, Israel; Adi.Enoch-Levy@sheba.health.gov.il; 3 Beer-Yaacov and Ness Ziona Mental Health Center, Ness Ziona 70350, Israel; kapon.shirley@gmail.com; 4 Pediatric Psychosomatic Department, Safra Children’s Hospital, Sheba Medical Center, Tel Hashomer, Ramat Gan 52621, Israel; yaramit@netvision.net.il; 5 Faculty of Health Sciences, Division of Psychiatry, Ben Gurion University of the Negev, Beer Sheva 84101, Israel; wtztu@yahoo.com

**Keywords:** anorexia nervosa, anxiety, body image, bulimia nervosa, depression, dissociation, mediation, moderation, suicidality

## Abstract

In patients with eating disorders (EDs), elevated dissociation may increase the risk of suicide. Bodily related disturbances, depression, and anxiety may intervene in the association between dissociation and suicidality. In this study we aimed to examine the influence of bodily related disturbances, depression, anxiety, severity of ED symptoms, body mass index (BMI), and type and duration of the ED on the relationship between elevated dissociation and elevated suicidality. The study included 172 inpatients: 65 with anorexia nervosa restricting type, 60 with anorexia nervosa binge/purge type, and 37 with bulimia nervosa. Participants were assessed using self-rating questionnaires for dissociation, suicidality, bodily related parameters, and severity of ED symptomatology, depression, and anxiety. We found that dissociation and suicidality were directly associated. In addition, depression and anxiety moderated the mediating role of body image parameters in the association between increased dissociation and increased suicidality. Thus, only in inpatients with high depression and anxiety, i.e., above the median range, body image disturbances were found to mediate the association between dissociation and suicidality. ED-related parameters did not moderate these relationships. Our study demonstrates that in inpatients with EDs, increased dissociation may be significantly associated with increased suicidality, both directly and via the intervening influence of body image, depression, and anxiety.

## 1. Introduction

Eating disorders (EDs) represent severe psychiatric disorders, associated with considerable morbidity and reduced overall wellbeing [1]. The standardized mortality rate in patients with anorexia nervosa (AN) is five times greater than that of healthy controls [2], with suicide being a major cause of death [3]. Suicidal ideation has been found in 20–43% of patients with AN, and the risk of death by suicide is 18–31 times higher in AN compared with the general population [4]. Bulimia nervosa (BN) may also have an elevated risk of death compared with the general population, although to a lesser extent than AN [3]. While studies have been inconsistent regarding the risk of death by suicide in BN, suicide attempts and suicidal ideation have been steadily shown to be the most prevalent in BN among the entire spectrum of EDs [5].

Elevated suicidality in EDs has been linked to several factors, including a history of physical or sexual abuse [6,7], emotional dysregulation [8], comorbid psychiatric disturbances, primarily depression [9], and a shared genetic risk underlying the coexpression of AN, depression, and attempted suicide [10,11]. Although most evidence has linked suicidality to binge/purge ED symptoms [7,12], a recent study found that restrictive eating may also be associated with suicidal ideation in youth with low-weight EDs, above and beyond the influence of other maladaptive consummatory behaviors [13].

Orbach’s theory of suicide, centering around trauma, dissociation, and pain indifference, provides an appropriate framework for understanding suicidality in the context of EDs. According to Orbach [14], early intolerable traumatic events may lead to defensive dissociative reactions which, in turn, increase detachment from the body and tolerance of physical pain. Suicide attempts and non-suicidal self-injury (NSSI) are understood in this context as enhancing dissociation, leading to elevated pain indifference and bodily disregard [14]. These processes may increase the risk of suicidality in the presence of elevated mental pain [14]. Orbach’s theory of suicide seems particularly relevant to EDs, as it emphasizes the importance of problematic attitudes toward the body, a key disturbance in EDs [15,16]. The other main factors in Orbach’s theory, early trauma and dissociation, are also highly prevalent in patients with EDs [17,18].

The modern diagnostic criteria of dissociation include a disturbance or change in the usually integrative functions of memory, identity, or consciousness [19]. Dissociation can be expressed in five different symptom groups: amnesia, depersonalization, derealization, mixed identity, and identity fragmentation [20]. There is often an association between dissociative phenomena and the existence of previous traumatic experiences in the person’s life [19,21]. These experiences may be associated with the development of a host of dissociative symptoms [20], resulting from both psychological and physiological derangements [22]. Research has also shown the importance of the relationship between dissociation and previous traumatic experiences [18] in patients with EDs [18,21,23]. Specifically, it is the dissociative-related experiences of feeling detached from one’s body, emotions, and cognition that have been repeatedly associated with an increase in binge-eating and purging behaviors [18].

In support of the relationship between dissociation and suicidality, a meta-analysis by Calati et al. [24] found that dissociative symptoms are associated with a greater risk of both attempted suicide and NSSI in patients with different psychiatric disturbances. Dissociation from one’s body was found to be higher in suicide attempters in comparison to people with no history of suicide [25,26]. Moreover, in a recent study of clinically referred adolescents, increased suicidal risk was associated with dissociative symptoms, independent of the presence of borderline or affective symptoms [27]. Another study found an association between elevated risk of suicidal ideation/attempted suicide and dissociation, regardless of the extent of pain tolerance [28]. Last, elevated dissociative symptoms were also associated with attempted suicide and NSSI in patients with EDs [29].

Beyond dissociation, Orbach stressed that attitudes towards the body might affect suicidality, and indeed they were found to discriminate suicidal from non-suicidal adolescents [30]. Moreover, the capability to engage in suicidal behavior was significantly associated with attempted suicide when self-preservative investment in one’s body was low [31].

Some studies have addressed the relationship between body-related disturbances and suicidality in patients with EDs. Thus, positive appearance evaluation, bodily satisfaction, and body investment were lower in ED patients with a history of NSSI compared to those without NSSI [32]. In a non-clinical sample, ED symptoms and body dissatisfaction were associated with increased suicidal ideation in participants with high levels of disgust with themselves and the world around, but not in participants with low levels of disgust [33]. Last, Forrest et al. found that in patients with EDs, ED symptoms and body dissatisfaction were related to suicidal ideation via the mediating effect of higher burdensomeness [34].

Another model, the integrated motivational-volitional model of suicide, posits that the transition from distress to suicide ideation and from ideation to action is determined by state-specific factors that either facilitate or hinder movement between suicidal states [35]. Along these lines, the aim of the present study was to examine the relationship between dissociative symptoms and suicidality in female adolescents with EDs, and to assess possible factors that would intervene in these relationships. We hypothesized that: (1) Both dissociation and suicidality would be greater in ED patients with binge/purge type EDs vs. restricting type EDs. (2) Dissociation would have a direct effect on suicidality. (3) ED-related body image disturbances would mediate the relationship between dissociation and suicidality, so that more disturbed attitudes toward the body would increase the influence of dissociation on suicidality, hence the overall suicide risk, while positive attitudes toward the body would reduce the influence of dissociation on suicidality. (4) Relevant demographic and clinical variables, including the duration of ED, type of ED, body mass index (BMI), and the severity of depression, anxiety, and ED symptomatology would intervene (i.e., moderate) in the mediating influences of the body-related dimensions on the association between dissociation and suicidality.

## 2. Methods

### 2.1. Participants

The study included 172 female adolescents hospitalized with an ED between 2002 and 2016 in the Pediatric Psychosomatic Department at the Safra Children’s Hospital, Sheba Medical Center, Tel Hashomer, Israel. Seventy-five participants were diagnosed with anorexia nervosa restricting type (AN-R), 60 with anorexia nervosa binge/purge type (AN-B/P), and 37 with bulimia nervosa (BN).

Inclusion criteria were: female gender, age over 15, parents and patients agreeing to participate in the study, and having a good understanding of the Hebrew language. Patients were excluded if they had current or lifetime schizophrenic spectrum disorders (patients with these disorders are not hospitalized in this department), intellectual disability (these patients do not take part in research studies), or any medical disorder with the potential to affect food consumption and weight (e.g., diabetes mellitus, thyroid disorders).

### 2.2. Instruments

#### 2.2.1. Interviews

The diagnosis of an ED was established according to the DSM-IV criteria [36] using the Structured Clinical Interview for DSM-IV Axis I Disorders-Patient Edition SCID-I/P Version 2.0; [37]. For the purpose of this study, case files were reviewed and diagnoses were revised according to the DSM-5 criteria for EDs [38].

#### 2.2.2. Self-Rating Scales

Maladaptive-eating-related parameters were assessed using the 26-item Eating Attitude Test-26 (EAT-26) [39], previously shown to successfully differentiate Israeli patients with EDs from non-ED controls [16]. The internal consistency of the EAT-26 in the present study was α = 0.93.

For the purpose of this study, we also used two subscales: the Eating Disorders Inventory-2 (EDI-2) [40], the body dissatisfaction scale (EDI-2-BD), and the drive for thinness scale (EDI-2-DT) scale. The EDI-2 was previously shown to successfully differentiate Israeli patients with EDs from non-ED controls [15]. The internal consistency of the EDI-2-BD in the present study was α = 0.94, and that of the EDI-2-DT was α = 0.88.

Dissociative symptoms were assessed using the 27-item Perceptual Alteration Scale (PAS) [41]. The scores for each item range from 1 (low dissociation) to 4 (high dissociation. Examples of PAS items include: “I see myself different than other people see me”; even if I skip meals, I do not feel hungry”; “I feel disconnected from my body”; “I have uncontrolled outbursts of laughing and crying.” Higher scores indicate greater dissociation. The PAS items converge into three factors assessing dissociation-related modifications in the subjective experiences of emotion, control, and cognition. The general reliability analysis of the PAS yielded an alpha score of 0.95 [41].

In her initial study of 114 students, Sanders found that the 40 students with binge eating behavior scored higher on the PAS than the other 74 students with normal eating [41]. In another study [42], the PAS differentiated patients with AN and BN from normal controls.

The PAS is particularly useful in the identification of dissociation-related emotional symptoms, since it has been found to be significantly related to depression, anxiety, and emotional abuse in community controls and patients with psychiatric disturbances, including EDs [21]. Thus, using the PAS, EDs and eating disturbances have been related to emotional dissociation and dissociation in general [43].

In the Israeli adaptation of this scale [44], the internal consistency of each of the three factors of the PAS was high and, therefore, three general scores were computed by averaging the item scores of each factor [44]. Similarly, in the present study, three factors were extracted using a factor analysis, i.e., dissociation of affect, control, and cognition. The internal consistency of the PAS scales in our study was α = 0.86 for dissociation of affect, α = 0.79 for control, α = 0.66 for cognition, and α = 0.92 for the total PAS score, derived from the addition of the three separate subscales.

Suicidal tendencies were assessed using the Multi Attitudes Suicide Tendencies (MAST) Scale [45]. This 30-item scale provides four independent scores: attraction to life (AL), repulsion by life (RL), attraction to death (AD), and repulsion by death (RD). Low AL, high RL, high AD, and low RD reflect high suicidal tendencies. In this study, a total score has been calculated, with the AL and RD scores reversed, with a higher score indicating greater suicidal tendencies. The validity of the MAST in discriminating suicidal from non-suicidal adolescents and its test-retest reliability was determined previously [30]. Elevated suicidal tendencies according to the MAST were previously found in patients with EDs in comparison to controls [16]. In the present study, the internal consistency of the four MAST scales was α = 0.88 for AL, α = 0.84 for AD, α = 0.77 for RL, and α = 0.92 for RD. The internal consistency for the total score was α = 0.58.

Body-related attitudes and feelings have been measured using the Body Investment Scale (BIS) [46]. This 24-item scale includes four factors: attitudes and feelings toward the body, comfort in touch, body care, and body protection. Previous studies have shown that the BIS differentiates between suicidal and non-suicidal adolescents [30,46]. A less favorable attitude toward the body according to the BIS has been previously found in patients with EDs in comparison to controls [16]. In the present study, the internal consistency of the BIS was α = 0.86 for attitudes and feelings toward the body, α = 0.75 for comfort in touch, α = 0.73 for body care, and α = 0.72 for body protection. The internal consistency for the total score in this study was α = 0.85.

The Contour Drawing Rating Scale (CDRS) [47], consisting of nine female contour images sorted from highly underweight to highly overweight, is used to assess body image. The CDRS has been found to show good validity and test-retest reliability r = 0.78 [47], and is accepted as a standardized tool for the assessment of body image disturbances in clinical and community populations [47,48]. The CDRS has been previously shown to distinguish Israeli patients with EDs from healthy controls [15]. In the present study, the internal consistency of the CDRS was α = 0.76.

Depression was assessed using the 21-item Beck Depression Inventory (BDI) [49] previously used in patients with EDs [50], including in Israeli samples [51]. The internal consistency of the BDI in the present study was α = 0.85.

Anxiety was assessed using the 40-item State-Trait Anxiety Inventory (STAI) [52], measuring the severity of anxiety at the time of examination (STAI-State; STAI-S) and the general tendency to display anxiety (STAI-Trait; STAI-T). The STAI was previously used in patients with EDs [50], including in Israeli samples [16,51]. The internal consistency of the STAI-S in the present study was α = 0.78, and of the STAI-T, α = 0.80.

### 2.3. Procedure

Participants and parents, in the case of minors under the age of 18, signed a written informed consent form, after being informed about the aims of the study. The study was approved by the Institutional Review Board of the Sheba Medical Center, Tel Hashomer, Israel. Upon admission, patients were independently interviewed by three experienced child and adolescent psychiatrists (D.S., A.Y., and A.E.L). The inter-rater reliability correlation between the three psychiatrists for the ED diagnoses was *r* = 0.92. Diagnoses were further confirmed in departmental clinical staff meetings. Only patients for whom there has been a unanimous agreement on their ED diagnosis could enter the study. The study’s self-report questionnaires were completed within two weeks of admission after the stabilization of the patients’ medical condition. They were distributed in the morning hours in a random order by BA or MA level psychology students. Demographic and clinical variables, including age, duration of illness, and length of inpatient treatment, were recorded using a demographic questionnaire and from the patients’ medical records. Weight and height were regularly taken in the morning hours according to standardized procedures [53]. Body mass index (BMI) was calculated as weight divided by height squared [54].

### 2.4. Statistical Analysis

First, descriptive statistics were produced using frequencies for categorical variables and means with standard deviations for continuous variables.

Differences between patients with AN-R, AN-B/P, and BN in demographic, clinical, and psychometric variables were assessed using a multivariate analysis of variance (MANOVA). Post hoc comparisons were conducted using Hochberg correction for the cases where the variances were equal and Tamhane correction when the variances were not equal. Correlations between the different variables were computed using Pearson correlation coefficients.

To assess the mediation relationships between the different study variables, a structural equation modelling (SEM) has been conducted. The following indices have been used to evaluate the model: chi-squared, which is acceptable when the value is not significant; the goodness of fit index (GFI), the comparative fit index (CFI), the non-normed fit index (NNFI), (adequate values—above 0.90, excellent fit—above 0.95), and the root mean square error of approximation (RMSEA) (adequate values—less than 0.08, excellent fit—less than 0.06) [55]. The models have been controlled for age. Level of significance (*p*-value) has been put at 5%. Data have been entered and analyzed using SPSS version 26 and AMOS version 25.

## 3. Results

Table 1 summarizes the differences between patients with AN-R, AN-B/P, and BN for the psychometric, demographic, and clinical variables. Regarding the demographic and clinical characteristics, there was a significant between-group difference in the age (*F*(2, 166) = 3.73, *p* = 0.03, *η*^2^ = 0.04). Specifically, patients with AN-R (M = 15.87, SD = 1.45) were younger in comparison with the BN patients (M = 16.60, SD = 0.99; *p* < 0.01). Moreover, there was a significant between-group difference in the BMI (*F*(2, 166) = 73.56, *p* < 0.01, *η*^2^ = 0.47). Specifically, patients with BN (M = 21.97, SD = 4.08) had greater BMI in comparison with the patients with AN-R (M = 16.26, SD = 1.58; *p* < 0.01) and the AN-B/P patients (M = 16.94, SD = 1.73; *p* < 0.01). In addition, there was a significant between-group difference in illness duration (*F*(2, 166) = 3.20, *p* = 0.04, *η*^2^ = 0.04). However, in post hoc pairwise comparisons, no significant results were found between the groups.

Between-group differences were found for all psychometric variables except for the two STAI dimensions Thus, a significant between-group difference was found for the PAS (*F*(2, 166) = 12.88, *p* < 0.01, *η*^2^ = 0.10). Specifically, patients with AN-R (M = 57.05, SD = 14.16) showed fewer dissociative symptoms in comparison with patients with AN-B/P (M = 66.22, SD = 16.05, *p* < 0.01) and BN (M = 67.06, SD = 12.88, *p* < 0.01) (see Table 1).

Second, a significant between-group difference was found for the MAST (*F*(2, 166) = 4.62, *p* < 0.01, *η*^2^ = 0.05). Specifically, patients with AN-R (M = 2.33, SD = 0.63) had lower suicidality, in comparison with the AN-B/P group (M = 2.70, SD = 0.80, *p* < 0.01) (see Table 1).

Third, a significant between-group difference was found in the investment in the body as measured with the BIS (*F*(2, 166) = 4.79, *p* < 0.01, *η*^2^ = 0.05). Specifically, patients with AN-R (M = 3.08, SD = 0.54) had higher scores on the BIS than patients with AN-B/P (M = 2.79, SD = 0.62, *p* = 0.01) (See Table 1).

Fourth, a significant between-group difference was found for the CDRS scores (*F*(2, 166) = 9.31, *p* < 0.01, *η*^2^ = 0.10). Specifically, patients with AN-R (M = 2.69, SD = 2.88) scored lower on the CDRS, i.e., regarded themselves as less overweight than patients with AN-B/P (M = 4.38, SD = 2.69, *p* < 0.01) and BN (M = 4.82, SD = 2.60, *p* < 0.01) (see Table 1).

In addition, significant between-group differences were found for the two EDI-2 scales included in our study, drive for thinness (EDI-2-DT; *F*(2, 166) = 5.56, *p* < 0.01, *η*^2^ = 0.06) and body dissatisfaction (EDI-2-BD; *F*(2, 166) = 8.18, *p* < 0.01, *η*^2^ = 0.09). Specifically, patients with AN-R (M = 12.89, SD = 7.07) had lower scores (i.e., less disturbance) on the EDI-2-DT in comparison to patients with AN-B/P (M = 16.02, SD = 5.73, *p* = 0.02) and BN (M = 16.26, SD = 4.96, *p* = 0.02), and lower scores on the EDI-2-BD than patients with AN-B/P (M = 20.85, SD = 8.37, *p* < 0.01) and B/N (M = 21.15, SD = 5.98, *p* < 0.01) (See Table 1).

Moreover, a significant between-group difference was found for depression (BDI; *F*(2, 166) = 6.38, *p* < 0.01, *η*^2^ = 0.07). Specifically, patients with AN-B/P (M = 37.03, SD = 15.25) had more severe depression symptoms in comparison with AN-R patients (M = 28.63, SD = 12.77, *p* < 0.01) and marginally elevated BDI scores in comparison to BN patients (M = 29.97, SD = 14.52, *p* = 0.06) (See Table 1).

Last, there was a significant between-group difference in eating-related pathology (EAT-26) (*F*(2, 166) = 6.89, *p* < 0.01, *η*^2^ = 0.08). Specifically, patients with AN-R (M = 40.20, SD = 17.95) had lower EAT-26 scores (i.e., lower eating-related pathology) than patients with AN-B/P (M = 50.50, SD = 18.66, *p* < 0.01; see Table 1).

Table 2 presents the descriptive statistics and correlations among the different study variables. Specifically, dissociation (total PAS score) was positively correlated with suicidality (MAST), the severity of eating-related pathology (EAT-26), body-image-related disturbances (CDRS, EDI-2-DT, and EDI-2-BD), depression (BDI), and anxiety STAI-S, STAI-T).

Suicidality (MAST) was negatively correlated with the investment in one’s body (BIS), and positively correlated with body-image-related disturbances (CDRS, EDI-2-DT, and EDI-2-BD), and with the severity of eating-related pathology (EAT-26), depression (BDI), and anxiety (STAI-S, STAI-T).

### 3.1. Mediation Model

**Figure 1:**
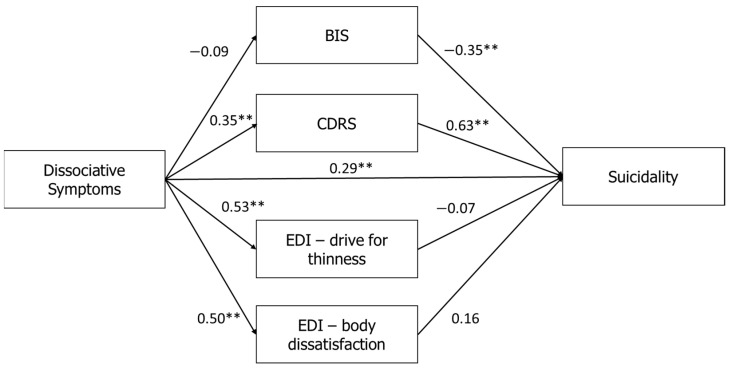
Mediation of the relationships between dissociative symptoms (PAS) and suicidality (MAST) using body image dimensions as mediators. Note: ** *p* < 0.01. PAS: Perceptual Alteration Scale; MAST: Multi Attitude Suicide Tendencies Scale; BIS: Body Investment Scale; CDRS: Contour Drawing Rating Scale; EDI-2: Eating Disorders Inventory-2.

We conducted a structural equation modeling (SEM) to assess the way in which body-related dimensions (BIS, CDRS, EDI-2-DT, and EDI-2-BD) mediate the relationship between dissociation (PAS) and suicidality (MAST). The findings are summarized in Figure 1. This model provided fair goodness of fit indices (*χ*^2^(3) = 31.42; *p* < 0.001; GFI = 0.95; NFI = 0.92; CFI = 0.92; RMSEA = 0.09). First, a direct effect was found between the PAS and the MAST (*β* = 0.29, *p* < 0.001). Second, the PAS was positively correlated with the CDRS (*β* = 0.35, *p* < 0.001), EDI-2-DT (*β* = 0.53, *p* < 0.001), and EDI-2-BD (*β* = 0.50, *p* < 0.001). Examination of the relationships between the mediators and the MAST showed a negative correlation with total BIS (*β* = −0.35, *p* < 0.01), and a positive correlation with the CDRS (*β* = 0.63, *p* < 0.01). To summarize, our findings showed a partial mediation effect in the association between dissociation (PAS) and suicidality (MSAT), with an indirect mediation of body-related influences (*β* = 0.15, *p* < 0.01), in addition to a direct association between dissociation and suicidality (*β* = 0.29, *p* < 0.01). Specifically, less positive investment in the body (BIS) and perceiving the body as heavier (CDRS) mediated in increasing the influence of dissociation (PAS) on suicidality (MAST).

### 3.2. Moderation Effects

A moderation effect on the mediation of body-related dimensions in the association between dissociation (PAS) and suicidality (MAST) was found for depression (BDI (*β* = 0.21, *p* = 0.01). To probe this effect, we split the sample into individuals who scored lower than the median of BDI in this sample (Med = 31.0) and higher than the median BDI. Then, we calculated the SEM model for the two groups, yielding fair goodness of fit indices (*χ*^2^(6) = 40.01; *p* < 0.001; GFI = 0.94; NFI = 0.86; CFI = 0.88; RMSEA = 0.09).

**Figure 2:**
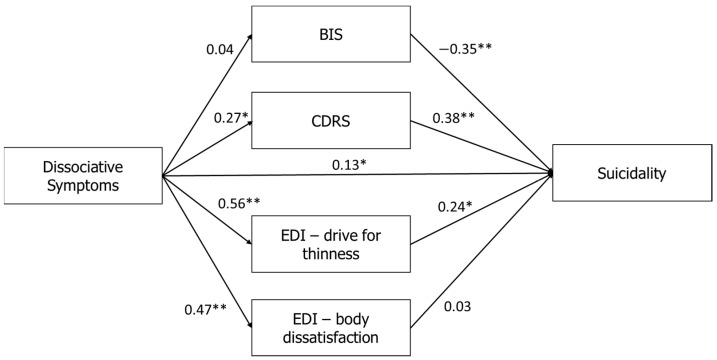
Relationships between dissociative symptoms (PAS) and suicidality (MAST) in the high-depression group when the mediators are the BIS, CDRS, EDI-2-Drive for Thinness, and EDI-2-Body Dissatisfaction. Note: * *p* < 0.05, ** *p* < 0.01. PAS: Perceptual Alteration Scale; MAST: Multi Attitude Suicide Tendencies Scale; BIS: Body Investment Scale; CDRS: Contour Drawing Rating Scale; EDI-2: Eating Disorders Inventory-2.

Overall, stronger correlations were found in the model of high-BDI patients (see Figure 2). Specifically, a direct effect between the PAS and the MAST (*β* = 0.34, *p* < 0.001) was found only for high-depression patients (BDI > 31). In addition, the PAS was positively correlated in this group with heavier body perception on the CDRS (*β* = 0.27, *p* < 0.001), and with higher scores on the EDI-2-DT (*β* = 0.56, *p* < 0.001) and EDI-2-BD (*β* = 0.47, *p* < 0.001). For suicidality, we found that the total BIS was negatively correlated with the MAST (*β* = −0.35, *p* < 0.01), while the CDRS was positively correlated with the MAST (*β* = 0.38, *p* < 0.01; see Figure 2).

**Figure 3:**
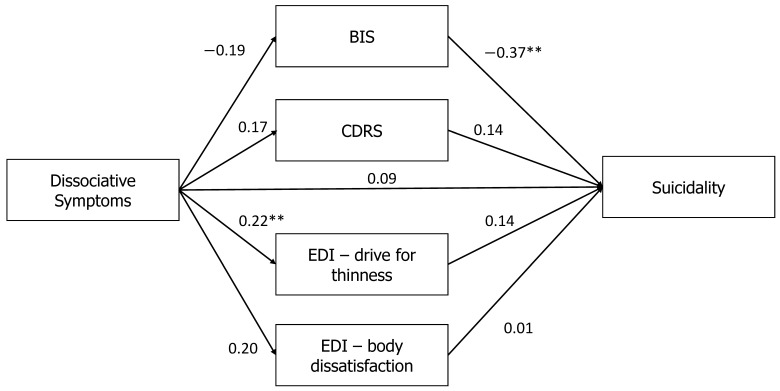
Relationships between dissociative symptoms (PAS) and suicidality (MAST) in the low-depression group in Table 2. Drive for Thinness, and EDI-2-Body Dissatisfaction. Note: ** *p* < 0.01. PAS: Perceptual Alteration Scale; MAST: Multi Attitude Suicide Tendencies Scale; BIS: Body Investment Scale; CDRS: Contour Drawing Rating Scale; EDI-2: Eating Disorders Inventory-2.

To summarize, examination of the mediation between the PAS and MAST showed a full mediation effect for the high-depression group, with an indirect effect (*β* = 0.13, *p* < 0.01). Specifically, elevated depression increased the mediating effects of maladaptive body-related attitudes and perceptions on the association between dissociation and suicidality. By contrast, no mediation effect was found for the low-depression group (*β* = 0.04, *p* = 0.82; see Figure 3).

A moderation effect on the mediation of body-related dimensions in the association between dissociation and suicidality was also found for trait anxiety (STAI-T; *β* = 0.62, *p* < 0.01). To probe this effect, we split the sample into individuals who scored lower than the median of STAI-T in this sample (Med = 55.50) and higher than the median STAI-T. Then, we calculated the SEM model for both groups, yielding fair goodness of fit indices (*χ*^2^(6) = 34.01; *p* < 0.001; GFI = 0.94; NFI = 0.90; CFI = 0.91; RMSEA = 0.16).

**Figure 4:**
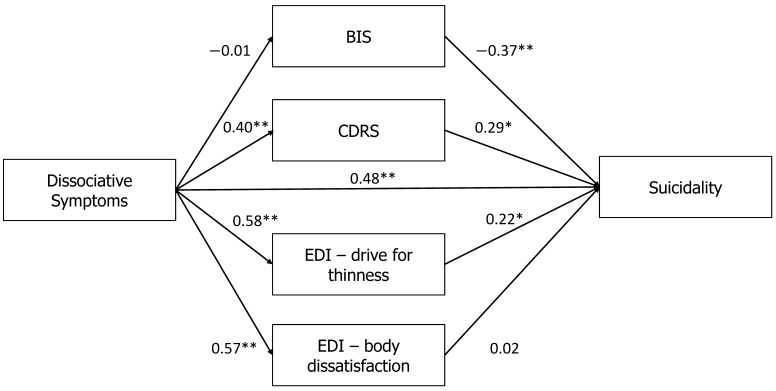
Relationships between dissociative symptoms (PAS) and suicidality (MAST) in the high-trait-anxiety group when the mediators are the BIS, CDRS, EDI-2-Drive for Thinness, and EDI-2-Body Dissatisfaction. Note: * *p* < 0.05, ** *p* < 0.01. BIS: Body Investment Scale; CDRS: Contour Drawing Rating Scale; EDI-2: Eating Disorders Inventory-2.

Overall, stronger correlations were found in the model of high- anxiety-trait patients (see Figure 4). Specifically, only for high-anxiety-trait patients, a direct effect was between the PAS and the MAST (*β* = 0.48, *p* < 0.001). In addition, the PAS was positively correlated with heavier body perception on the CDRS (*β* = 0.40, *p* < 0.001), and with higher scores on the EDI-2-DT (*β* = 0.58, *p* < 0.001) and the EDI-2-BD (*β* = 0.57, *p* < 0.001). For suicidality, we found that total BIS was negatively correlated with the MAST (*β* = −0.37, *p* < 0.01), while the CDRS (*β* = 0.29, *p* < 0.01) and EDI-2-DT were positively correlated with the MAST (*β* = 0.22, *p* < 0.05).

**Figure 5:**
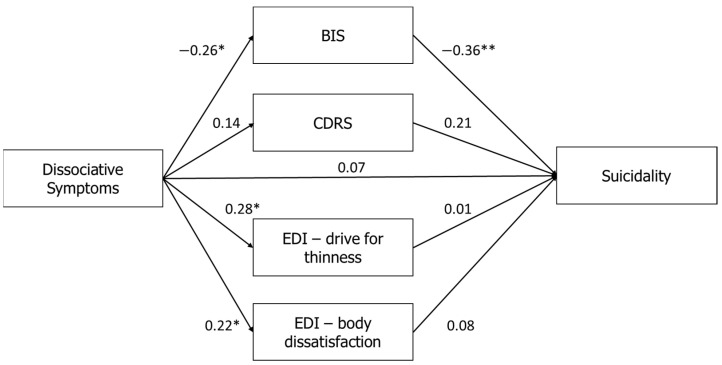
Relationships between dissociative symptoms (PAS) and suicidality (MAST) in the low-trait anxiety group when the mediators are the BIS, CDRS, EDI-2-Drive for Thinness, and EDI-2-Body Dissatisfaction. Note: * *p* < 0.05, ** *p* < 0.01. PAS: Perceptual Alteration Scale; MAST: Multi Attitude Suicide Tendencies Scale; BIS: Body Investment Scale; CDRS: Contour Drawing Rating Scale; EDI-2: Eating Disorders Inventory-2.

To summarize, examination of the mediation between the PAS and the MAST showed a full mediation effect for the high STAI-T group, with an indirect effect (*β* = 0.14, *p* < 0.01); specifically, elevated trait anxiety increased the mediating effects of maladaptive body-related attitudes and perceptions on the association between dissociation and suicidality. By contrast, no mediation effect was found for the low STAI-T group (*β* = 0.01, *p* = 0.93; see Figure 5).

Last, no moderation effect was found for the STAI-S (*β* = 0.11, *p* = 0.48), EAT-26 (*β* = 0.06, *p* = 0.75), duration of illness (*β* = −0.04, *p* = 0.72), BMI (*β* = −0.09, *p* = 0.31), or type of ED diagnosis (*β* = 0.09, *p* = 0.75). No moderation effect was examined for age due to low variance at age in the sample.

## 4. Discussion

The aims of the current study were to investigate the relationship between dissociation and suicidality in female adolescent patients with EDs, and to explore potential factors that mediate these relationships, as well as other factors that, in their turn, moderate these mediational processes.

As expected, confirming our first hypothesis, dissociative symptoms as well as suicidality were higher in binge-purge EDs, i.e., AN B/P (for both dimensions) and BN (only for the PAS). These results replicate findings from previous studies in EDs, showing that both dissociation and attempted suicide/NSSIs/suicidal thoughts are more prevalent in AN-B/P and BN [18,56,57]. However, most other psychometric variables, except for anxiety, were also more pronounced in patients with B/P type EDs in comparison to AN-R (AN-B/P all variables, BN-CDRS, EDI-2-BD, and EDI-2-DT). These findings have also been shown elsewhere [58,59,60], and are likely related to a greater severity of illness in AN-B/P, and to an overall greater emotional dysregulation and impulsivity in the binge/purge subtype in general [61]. Dissociation can be, in this respect, conceptualized as one regulatory means to reduce emotional dysregulation and impulsivity, and elevated suicidal risk is a failure of this regulation. Nonetheless, it is of note that the type of ED had no moderating influence on the mediation of body-related parameters in the relation between dissociation and suicidality.

In keeping with our second hypothesis, we found a direct effect of dissociation on suicidality. Furthermore, we found that dissociation and suicidality were both associated with body image parameters (CDRS, EDI-2, and EDI-BD). First, the PAS was positively correlated with the CDRS, EDI-2-DT, and EDI-2-BD. This suggests that dissociation may impair accurate perception of the body in patients with EDs (perceiving oneself as heavier on the CDRS), leading in turn to greater dissatisfaction with the body, and hence, to greater drive to lose weight. The association between body perception disturbances and body dissatisfaction is a well-known finding in EDs [62]. Similarly, dissociative features might play a fundamental role in producing body image distortions in patients with EDs [63].

Second, the CDRS was positively correlated with suicidality, suggesting that body-perception disturbance in seeing oneself as heavier had the potential to increase suicidal risk. Although there was an inherent difference in the BMI of patients with AN vs. BN, with both AN-R and AN-B/P patients having a lower BMI than patients with BN, we found that not only patients with BN, but also patients with AN-B/P perceived themselves as heavier than patients with AN-R. Nonetheless, it is of note that the scales used in our study, the EDI-2-BD, EDI-2-DT, and CDRS, might not be sensitive enough to discern the body misperception or the poor insight related to body perception in patients with AN [64].

Moreover, BMI had no moderating effect on the mediation of body-related parameters in the relationships between dissociation and suicidality. Altogether, our findings suggest that it is the ED patients’ perception of their weight, rather than their actual weight, that might increase their dissatisfaction with their body [65], and, in turn, their overall risk for suicide [34].

By contrast, positive attitudes and behaviors toward the body, as assessed with the BIS, were negatively correlated with suicidality. This suggests that a positive investment in the body, independent of body-size perception and body image distortion, might serve to protect against suicidal behaviors. It is interesting to note, in this respect, that whereas dissociation was associated only with body perception disturbance and body dissatisfaction, suicidality was associated also with a more comprehensive, non-ED-related construct of protective vs. aversive attitudes and behaviors toward the body.

To summarize, our third hypothesis, based on Orbach’s [14] theory of suicide, suggesting that in patients with EDs, positive attitudes and behaviors toward the body would reduce the effect of dissociation on suicidality while negative attitudes and behaviors toward the body would increase suicidality, has been partly confirmed (partial mediation effect). This replicates earlier findings both in adolescents in general [30,46] and in adolescents with EDs [16]. In this respect, our results point to the importance of promoting non-bodily perception related aspects in the treatment of EDs. This can be achieved, perhaps, by using holistic integrative therapy strategies such as dialectical behavior therapy, mindfulness, relaxation, and/or meditation [66,67]. Nonetheless, body-perception-targeted treatments such as body exposure using a mirror, video, or computer-based feedback [15,66], are just as important. These complementary treatment strategies may be added to weight and disordered eating restoration to correct the impairments in body perception and overall bodily related attitudes in the necessary, yet burdensome process of accepting the new weight.

The study of the moderating effects of depression and anxiety on the mediating role of body image in the association between dissociation and suicidality is of relevance because comorbid depressive and anxiety disorders are highly prevalent in EDs [68,69]. Depression and anxiety may intensify ED-related pathology [9], as well as affect many non-eating psychopathologies in EDs, including dissociation [70], bodily related disturbances [71], and suicidality [9,10,11]. It is of note that our fourth hypothesis regarding the factors intervening in the mediation of the association between dissociation and suicidality has been supported for depression and trait anxiety, but not for ED symptom severity, BMI, duration of illness, or type of ED diagnosis.

In our study, for the high-depression and high-trait-anxiety groups only, in addition to a direct association between the PAS and the MAST, body image disturbances (CDRS, EDI-2-BD, EDI-2-DT) increased the influence of dissociation on suicidality, while positive investment in the body decreased this mediational effect (see Figure 2 and Figure 4). It is of note that the ED patients in our study were highly depressed (BDI score of the entire sample 31.71 ± 14.41, and of all groups around 30, see Table 1 and Table 2; according to Beck [49], a BDI score ≥ 30 signifies severe depression). Other studies of adolescent inpatients with EDs also found BDI scores around 30 [72,73]. These high BDI levels are likely associated with the high rates of comorbid depression in inpatients with EDs, the considerable malnutrition likely present in inpatients, and the reluctance of many adolescents to undergo inpatient treatment (although all patients in our department agreed to be hospitalized). The same holds true also for the STAI-T, with a score range of 20–80 (higher scores indicate more severe anxiety) and a cutoff point of 39–40 to detect clinical symptoms of anxiety [74]. The mean score of the STAI-T in our study (55.61 ± 11.43), likely represents severe state anxiety.

The results of our study emphasize the importance of treating the patients’ severe depression and anxiety [75]. We suggest that the management of both disturbances, whether with cognitive behavioral therapy [71], or serotonin specific reuptake inhibitors [76] might reduce the influence of dissociation on suicidality in patients with EDs, whether directly, or indirectly, via the mediation of body-related parameters. This consideration is of particular relevance for ED patients with sexual-trauma-related complex post-traumatic symptoms, showing specifically high levels of both dissociation and suicidality [77,78]. The lack of moderating effect of all ED-related parameters included, when bearing in mind the mediational role of all body-related parameters, may suggest that in line with Orbach’s [14] theory of suicide, bodily related disturbances may be of particular relevance in increasing the suicidal risk of patients with EDs, regardless of their malnutrition condition, severity of ED symptomatology, and duration of their illness. Nevertheless, as our sample consists only of inpatients with severe EDs, it might not be diverse enough to demonstrate a potential moderating effect of ED-related parameters on the associations between dissociation and suicidality.

In summary, according to the findings of our study and in line with Orbach’s [14] theory of suicide, bodily related disturbances might increase the suicidal risk of patients with EDs in the context of elevated dissociation, alongside the influence of additional severe comorbid depressive and/or anxiety symptoms.

### Limitations and Strengths

Several limitations of our study should be addressed. First, we relied on indirect measurement of suicidal tendencies with the MAST, rather than assessing actual suicidal behavior. Nonetheless, the use of this indirect method, significantly associated with increased suicidality in psychiatric patents in general [30] and in patients with EDs in particular [16], might decrease the risk of under-reported suicidal risk in our patients. Second, we did not check for a history of traumatic experiences, which are a key factor in Orbach’s theory of suicide, as well as in the development of dissociation. Third, our cross-sectional design allowed for the assessment of the associations between dissociation, body image, depression, anxiety, and suicidality, but did not enable us to evaluate the directionality of the influence of the parameters assessed in actually increasing the patients’ suicidal risk. Fourth, given our limited sample size, we used the total scores for the BIS and MAST measures rather than assessing, in addition, the potential influence of their specific sub-constructs (e.g., MAST attraction to life or BIS-body protection). The use of larger samples could assist in the investigation of these components and their respective contribution to our model. Fifth, the potential of the BDI and STAI-T to moderate the mediation of body-related parameters in the relationship between dissociation and anxiety was achieved only in patients with severe depressive and anxiety symptoms. Thus, less severe depression and anxiety might not have moderated this mediating effect of body image parameters. Sixth, we assessed our patients when they were acutely ill, not being able to disentangle the influence of malnutrition on our findings. In addition, we did not include a control group to validate the findings on our scales against non-ED populations. Last, as we studied only inpatients, our findings cannot be generalized to patients with less severe EDs.

Our study has, nevertheless, some important advantages. It is a hypothesis-generated research, based on a relatively large number of participants, and includes validated assessment tools. We have also assessed the patients’ weight and height, rather than relying on self-reports. We have specifically chosen the PAS for the assessment of dissociation, because the researchers developing this scale have specifically used it for patients with EDs [41,42]. Thus, it contains items related to dissociation of the body, emotions, and control, all of considerable relevance in the study of dissociation [18] and suicidality [32,79] in patients with EDs.

## 5. Conclusions

The aim of this study was to examine in ED inpatients the influence of bodily related disturbances, depression, anxiety, severity of ED symptoms, body mass index (BMI), and type and duration of the ED on the relationship between elevated dissociation and elevated suicidality. We found that dissociation and suicidality were directly associated. In addition, depression and anxiety moderated the mediating role of body image parameters in the association between increased dissociation and increased suicidality. Thus, only in inpatients with increased depression and anxiety, were body image disturbances found to mediate in the association between dissociation and suicidality. ED-related parameters did not moderate in these relationships.

Future studies should attempt to include information about actual suicidal behaviors and trauma history to verify and extend the findings of our model in a prospective longitudinal assessment of ambulatory patients with EDs throughout the course of their illness.

## Figures and Tables

**Table 1:** Differences in psychometric variables by eating disorder diagnosis.

	AN-R (N = 75)		AN-B/P (N = 60)		BN (N = 34)		F	*p*	*η* ^2^
	M	SD	M	SD	M	SD			
PAS	57.05	14.16 ^a^	66.22	16.05 ^b^	67.06	12.88 ^b^	8.77	<0.01	0.10
MAST	2.33	0.63 ^a^	2.70	0.80 ^b^	2.55	0.75	4.62	<0.01	0.05
BIS	3.08	0.54 ^a^	2.79	0.62 ^b^	2.86	0.49 ^b^	4.79	<0.01	0.05
CDRS	2.69	2.88 ^a^	4.38	2.96 ^b^	4.82	2.60 ^b^	9.31	<0.01	0.10
EDI-2-drive for thinness	12.89	7.07 ^a^	16.02	5.73 ^b^	16.26	4.96 ^b^	5.56	<0.01	0.06
EDI-2-body dissatisfaction	15.71	9.28 ^a^	20.85	8.37 ^b^	21.15	5.98 ^b^	8.18	<0.01	0.09
BDI	28.63	12.77 ^a^	37.03	15.25 ^b^	29.91	14.52 *	6.38	<0.01	0.07
STAI-S	54.88	11.56	56.97	13.18	57.21	11.21	0.68	0.51	-
STAI-T	54.35	10.50	57.37	12.20	57.18	8.05	1.58	0.21	-
EAT-26	40.20	17.95 ^a^	50.50	18.66 ^b^	47.47	16.05	6.89	<0.01	0.08
Age	15.87	1.45	16.28	1.42	16.60	0.99	3.73	0.03	0.04
BMI	16.26	1.58	16.94	1.73	21.97	4.08	73.56	<0.01	0.47
Disease duration (years)	0.26	0.79	0.23	0.89	0.72	1.40	3.20	0.04	0.04

Note: AN-R: anorexia nervosa restricting type; AN-B/P: anorexia nervosa binge/purge type; BN: bulimia nervosa; PAS: Perceptual Alteration Scale; MAST: Multi Attitude Suicide Scale; BIS: Body Investment Scale; CDRS: Contour Drawing Rating Scale; EDI-2: Eating Disorders Inventory-2; BDI: Beck Depression Inventory; STAI-S: State Trait Anxiety Inventory-State; STAI-T: State Trait Anxiety Inventory-T; EAT-26: Eating Disorder Inventory-26. BMI: Body Mass Index. Results with different superscripts indicate a significant difference in the findings in that row. Results with no superscripts indicate no differences from the other scores in that row. Results with an * indicate marginal differences.

**Table 2:** Descriptive statistics and correlations among the different study variables.

	M	SD	1	2	3	4	5	6	7	8	9	10	11	12
1. PAS	62.26	15.22												
2. MAST	2.49	0.74	0.45 **											
3. BIS	3.10	2.29	−0.09	−0.23 **										
4. CDRS	3.82	3.05	0.35 **	0.39 **	0.18 *									
5. EDI-2-DT	14.58	6.41	0.53 **	0.36 **	−0.21 **	0.44 **								
6. EDI-2-BD	18.61	8.67	0.50 **	0.46 **	−0.12	0.68 **	0.69 **							
7. BDI	31.71	14.41	0.60 **	0.62 **	−0.20 **	0.40 **	0.53 **	0.58 **						
8. STAI-S	55.69	12.65	0.36 **	0.48 **	−0.39 **	0.24 **	0.46 **	0.43 **	0.45 **					
9. STAI-T	55.61	11.43	0.49 **	0.53 **	−0.45 **	−0.24 **	0.52 **	0.46 **	0.51 **	0.79 **				
10. EAT-26	46.23	20.73	0.44 **	0.20 **	0.38 **	0.49 **	0.60 **	0.47 **	0.43 **	0.15 *	0.16 *			
11. Age	16.15	1.39	0.9	0.01	−0.00	0.06	0.13	0.09	0.05	−0.08	−0.01	0.11		
12. BMI	17.72	3.62	0.11	0.07	−0.03	0.28 **	0.15	0.23 **	−0.02	0.09	0.13	−0.03	0.09	
13. Disease duration (years)	0.34	0.98	0.01	−0.05	−0.01	−0.05	0.05	0.03	−0.11	0.00	−0.01	0.09	0.11	0.19 *

Note: * *p* < 0.05, ** *p* < 0.01; PAS: Perceptual Alteration Scale; MAST: Multi Attitude Suicide Scale; BIS: Body Investment Scale; CDRS: Contour Drawing Rating Scale; EDI-2-DT: Eating Disorders Inventory-2-Drive for thinness; EDI-2-BD: Eating Disorders Inventory-2-Body Dissatisfaction; BDI: Beck Depression Inventory; STAI-S: State Trait Anxiety Inventory-State; STAI-T: State Trait Anxiety Inventory-T; EAT-26: Eating Disorder Inventory-26. BMI: Body Mass Index.

## Data Availability

Data generated or analyzed in the study are available from the authors upon reasonable request.

## References

[1] Treasure J., Duarte T.A., Schmidt U. (2020). Eating Disorders. Lancet.

[2] Himmerich H., Hotopf M., Shetty H., Schmidt U., Treasure J., Hayes R.D., Stewart R., Chang C.-K. (2019). Psychiatric Comorbidity as a Risk Factor for Mortality in People with Anorexia Nervosa. Eur. Arch. Psychiatry Clin. Neurosci..

[3] Smink F.R.E., van Hoeken D., Hoek H.W. (2013). Epidemiology, Course, and Outcome of Eating Disorders. Curr. Opin. Psychiatry.

[4] Smith A.R., Zuromski K.L., Dodd D.R. (2018). Eating Disorders and Suicidality: What We Know, What We Don’t Know, and Suggestions for Future Research. Curr. Opin. Psychol..

[5] Kostro K., Lerman J.B., Attia E. (2014). The Current Status of Suicide and Self-Injury in Eating Disorders: A Narrative Review. J. Eat. Disord..

[6] Favaro A., Ferrara S., Santonastaso P. (2007). Self-Injurious Behavior in a Community Sample of Young Women: Relationship With Childhood Abuse and Other Types of Self-Damaging Behaviors. J. Clin. Psychiatry.

[7] Favaro A., Santonastaso P. (1997). Suicidality in Eating Disorders: Clinical and Psychological Correlates. Acta Psychiatr. Scand..

[8] Rania M., Monell E., Sjölander A., Bulik C.M. (2020). Emotion Dysregulation and Suicidality in Eating Disorders. Int. J. Eat. Disord..

[9] Fennig S., Hadas A. (2010). Suicidal Behavior and Depression in Adolescents with Eating Disorders. Nord. J. Psychiatry.

[10] Wade T.D., Fairweather-Schmidt A.K., Zhu G., Martin N.G. (2015). Does Shared Genetic Risk Contribute to the Co-Occurrence of Eating Disorders and Suicidality?. Int. J. Eat. Disord..

[11] Thornton L.M., Welch E., Munn-Chernoff M.A., Lichtenstein P., Bulik C.M. (2016). Anorexia Nervosa, Major Depression, and Suicide Attempts: Shared Genetic Factors. Suicide Life-Threat. Behav..

[12] Stein D., Lilenfeld L.R.R., Wildman P.C., Marcus M.D. (2004). Attempted Suicide and Self-Injury in Patients Diagnosed with Eating Disorders. Compr. Psychiatry.

[13] Wang S.B., Mancuso C.J., Jo J., Keshishian A.C., Becker K.R., Plessow F., Izquierdo A.M., Slattery M., Franko D.L., Misra M. (2020). Restrictive Eating, but Not Binge Eating or Purging, Predicts Suicidal Ideation in Adolescents and Young Adults with Low-Weight Eating Disorders. Int. J. Eat. Disord..

[14] Orbach I. (1994). Dissociation, Physical Pain, and Suicide: A Hypothesis. Suicide Life-Threat. Behav..

[15] Caspi A., Amiaz R., Davidson N., Czerniak E., Gur E., Kiryati N., Harari D., Furst M., Stein D. (2017). Computerized Assessment of Body Image in Anorexia Nervosa and Bulimia Nervosa: Comparison with Standardized Body Image Assessment Tool. Arch. Women’s Ment. Health.

[16] Stein D., Zinman D., Halevy L., Yaroslavsky A., Bachar E., Kreitler S., Orbach I. (2013). Attitudes toward Life and Death and Suicidality among Inpatient Female Adolescents with Eating Disorders. J. Nerv. Ment. Dis..

[17] Lyssenko L., Schmahl C., Bockhacker L., Vonderlin R., Bohus M., Kleindienst N. (2018). Dissociation in Psychiatric Disorders: A Meta-Analysis of Studies Using the Dissociative Experiences Scale. Am. J. Psychiatry.

[18] Vanderlinden J., Palmisano L., Seubert A., Virdi P. (2019). Trauma and Eating Disorders: The State of the Art. Trauma-Informed Approaches to Eating Disorders.

[19] van der Kolk B.A., van der Hart O. (1989). Pierre Janet and the Breakdown of Adaptation in psychological trauma. Am. J. Psychiatry.

[20] Gaon A., Kaplan Z., Dwolatzky T., Perry Z., Witztum E. (2013). Dissociative Symptoms as a Consequence of Traumatic Experiences: The Long-Term Effects of Childhood Sexual Abuse. Isr. J. Psychiatry Relat. Sci..

[21] Brack C.J., McCarthy C.J., Brack G., Hill M.B., Lassiter P.S. (2005). Usefulness of the Perceptual Alteration Scale. J. Prof. Couns. Pract. Theory Res..

[22] Krause-Utz A., Frost R., Winter D., Elzinga B.M. (2017). Dissociation and Alterations in Brain Function and Structure: Implications for Borderline Personality Disorder. Curr. Psychiatry Rep..

[23] Lev-ari L., Zohar A.H., Bachner-Melman R. (2021). Eating for Numbing: A Community-Based Study of Trauma Exposure, Emotion Dysregulation, Dissociation, Body Dissatisfaction and Eating Disorder Symptoms. PeerJ.

[24] Calati R., Bensassi I., Courtet P. (2017). The Link between Dissociation and Both Suicide Attempts and Non-Suicidal Self-Injury: Meta-Analyses. Psychiatry Res..

[25] Levinger S., Somer E., Holden R.R. (2015). The Importance of Mental Pain and Physical Dissociation in Youth Suicidality. J. Trauma Dissociation.

[26] Zoroglu S.S., Tuzun U., Sar V., Tutkun H., Savaçs H.A., Ozturk M., Alyanak B., Kora M.E. (2003). Suicide Attempt and Self-Mutilation among Turkish High School Students in Relation with Abuse, Neglect and Dissociation. Psychiatry Clin. Neurosci..

[27] Vine V., Victor S.E., Mohr H., Byrd A.L., Stepp S.D. (2020). Adolescent Suicide Risk and Experiences of Dissociation in Daily Life. Psychiatry Res..

[28] Rabasco A., Andover M.S. (2020). The Interaction of Dissociation, Pain Tolerance, and Suicidal Ideation in Predicting Suicide Attempts. Psychiatry Res..

[29] Demitrack M.A., Putnam F.W., Brewerton T.D., Brandt H.A., Gold P.W. (1990). Relation of Clinical Variables to Dissociative Phenomena in Eating Disorders. Am. J. Psychiatry.

[30] Orbach I., Stein D., Shani-Sela M., Har-Even D. (2001). Body Attitudes and Body Experiences in Suicidal Adolescents. Suicide Life-Threat. Behav..

[31] Brausch A.M., Nichols P., Laves E., Clapham R. (2021). Body Investment as a Protective Factor in the Relationship between Acquired Capability for Suicide and Suicide Attempts. Behav. Ther..

[32] Pérez S., Marco J.H., Cañabate M. (2018). Non-Suicidal Self-Injury in Patients with Eating Disorders: Prevalence, Forms, Functions, and Body Image Correlates. Compr. Psychiatry.

[33] Chu C., Bodell L.P., Ribeiro J.D., Joiner T.E. (2015). Eating Disorder Symptoms and Suicidal Ideation: The Moderating Role of Disgust. Eur. Eat. Disord. Rev..

[34] Forrest L.N., Bodell L.P., Witte T.K., Goodwin N., Bartlett M.L., Siegfried N., Eddy K.T., Thomas J.J., Franko D.L., Smith A.R. (2016). Associations between Eating Disorder Symptoms and Suicidal Ideation through Thwarted Belongingness and Perceived Burdensomeness among Eating Disorder Patients. J. Affect. Disord..

[35] O’Connor R.C. (2011). The Integrated motivational-Volitional Model of Suicidal Behavior. Crisis.

[36] American Psychiatric Association (1994). Diagnostic and Statistical Manual of Mental Disorders: DSM IV.

[37] First M.B., Spitzer R.L., Gibbon M., Williams J.B.W. (1996). Structured Clinical Interview for DSM-IV Axis I Disorders (Clinician Version).

[38] American Psychiatric Association (2013). Diagnostic and Statistical Manual of Mental Disorders: DSM-5.

[39] Garner D.M., Olmsted M.P., Bohr Y., Garfinkel P.E. (1982). The Eating Attitudes Test: Psychometric Features and Clinical Correlates. Psychol. Med..

[40] Garner D.M. (1991). Eating Disorder Inventory-2; Professional Manual.

[41] Sanders S. (1986). The Perceptual Alteration Scale: A Scale Measuring Dissociation. Am. J. Clin. Hypn..

[42] Sanders S., Boswell J., Hernandez J. A Study of Dissociation Contrasting Anorectics and Bulimics. Proceedings of the Third International Conference on Multiple Personality and Dissociative States.

[43] Rosen E.F., Petty L.C. (1994). Dissociative States and Disordered Eating. Am. J. Clin. Hypn..

[44] Orbach I., Mikulincer M., King R., Cohen D., Stein D. (1997). Thresholds and Tolerance of Physical Pain in Suicidal and Nonsuicidal Adolescents. J. Consult. Clin. Psychol..

[45] Orbach I., Milstein I., Har-Even D., Apter A., Tiano S., Elizur A. (1991). A Multi-Attitude Suicide Tendency Scale for Adolescents. Psychol. Assess..

[46] Orbach I., Mikulincer M. (1998). The Body Investment Scale: Construction and Validation of a Body Experience Scale. Psychol. Assess..

[47] Thompson M.A., Gray J.J. (1995). Development and Validation of a New Body-Image Assessment Scale. J. Personal. Assess..

[48] Lombardo C., Russo P.M., Lucidi F., Iani L., Violani C. (2004). Internal Consistency, Convergent Validity and Reliability of a Brief Questionnaire on Disordered Eating (DEQ). Eat. Weight Disord.-Stud. Anorex. Bulim. Obes..

[49] Beck A.T., Ward C.H., Mendelson M., Mock J., Erbaugh J. (1961). An Inventory for Measuring Depression. Arch. Gen. Psychiatry.

[50] Pollice C., Kaye W.H., Greeno C.G., Weltzin T.E. (1997). Relationship of Depression, Anxiety, and Obsessionality to State of Illness in Anorexia Nervosa. Int. J. Eat. Disord..

[51] Yackobovitch-Gavan M., Golan M., Valevski A., Kreitler S., Bachar E., Lieblich A., Mitrani E., Weizman A., Stein D. (2009). An Integrative Quantitative Model of Factors Influencing the Course of Anorexia Nervosa over Time. Int. J. Eat. Disord..

[52] Spielberger C.D., Gorsuch R.L., Lushene R.E. (1970). Manual for the State-Trait Anxiety Inventory.

[53] Tanner J., Kappy M.S., Blizzard R.M., Migeon C.J. (1994). Auxology. The Diagnosis and Treatment of Endocrine Disorders in Childhood and Adolescence.

[54] Bray G.A. (1992). Pathophysiology of Obesity. Am. J. Clin. Nutr..

[55] Arbuckle J.L. (2013). IBM SPSS Amos 22 User’s Guide.

[56] Longo P., Panero M., Amodeo L., Demarchi M., Abbate-Daga G., Marzola E. (2021). Psychoform and somatoform dissociation in anorexia nervosa: A systematic review. Clin. Psychol. Psychother..

[57] la Mela C., Maglietta M., Castellini G., Amoroso L., Lucarelli S. (2010). Dissociation in Eating Disorders: Relationship between Dissociative Experiences and Binge-Eating Episodes. Compr. Psychiatry.

[58] Carter J.C., Mercer-Lynn K.B., Norwood S.J., Bewell-Weiss C.V., Crosby R.D., Woodside D.B., Olmsted M.P. (2012). A Prospective Study of Predictors of Relapse in Anorexia Nervosa: Implications for Relapse Prevention. Psychiatry Res..

[59] Reas D.L., Rø Ø. (2018). Less Symptomatic, but Equally Impaired: Clinical Impairment in Restricting versus Binge-Eating/Purging Subtype of Anorexia Nervosa. Eat. Behav..

[60] DeJong H., Oldershaw A., Sternheim L., Samarawickrema N., Kenyon M.D., Broadbent H., Lavender A., Startup H., Treasure J., Schmidt U. (2013). Quality of Life in Anorexia Nervosa, Bulimia Nervosa and Eating Disorder Not-Otherwise-Specified. J. Eat. Disord..

[61] Kaye W. (2008). Neurobiology of Anorexia and Bulimia Nervosa. Physiol. Behav..

[62] Brown T.A., Shott M.E., Frank G.K.W. (2021). Body Size Overestimation in Anorexia Nervosa: Contributions of Cognitive, Affective, Tactile and Visual Information. Psychiatry Res..

[63] Demartini B., Nisticò V., Tedesco R., Marzorati A., Ferrucci R., Priori A., Gambini O., Caputo G.B. (2021). Visual Perception and Dissociation during Mirror Gazing Test in Patients with Anorexia Nervosa: A Preliminary Study. Eat. Weight Disord.-Stud. Anorex. Bulim. Obes..

[64] Phillipou A., Mountjoy R.L., Rossell S.L. (2017). Overvalued Ideas or Delusions in Anorexia Nervosa?. Aust. N. Z. J. Psychiatry.

[65] Fernández-Aranda F., Probst M., Meerman R., Vandereycken W. (1994). Body Size Estimation and Body Dissatisfaction in Eating Disorder Patients and Normal Controls. Int. J. Eat. Disord..

[66] Hartmann A.S., Thomas J.J., Greenberg J.L., Rosenfield E.H., Wilhelm S. (2015). Accept, Distract, or Reframe? An Exploratory Experimental Comparison of Strategies for Coping with Intrusive Body Image Thoughts in Anorexia Nervosa and Body Dysmorphic Disorder. Psychiatry Res..

[67] Pisetsky E.M., Schaefer L.M., Wonderlich S.A., Peterson C.B. (2019). Emerging Psychological Treatments in Eating Disorders. Psychiatr. Clin. N. Am..

[68] Casper R.C. (1998). Depression and Eating Disorders. Depress. Anxiety.

[69] Mattar L., Huas C., Duclos J., Apfel A., Godart N. (2011). Relationship between Malnutrition and Depression or Anxiety in Anorexia Nervosa: A Critical Review of the Literature. J. Affect. Disord..

[70] Gleaves D.H., Eberenz K.P. (1995). Correlates of Dissociative Symptoms among Women with Eating Disorders. J. Psychiatr. Res..

[71] Junne F., Zipfel S., Wild B., Martus P., Giel K., Resmark G., Friederich H.-C., Teufel M., de Zwaan M., Dinkel A. (2016). The Relationship of Body Image with Symptoms of Depression and Anxiety in Patients with Anorexia Nervosa during Outpatient Psychotherapy: Results of the ANTOP Study. Psychotherapy.

[72] Mekori E., Halevy L., Ziv S.I., Moreno A., Enoch-Levy A., Weizman A., Stein D. (2017). Predictors of Short-Term Outcome Variables in Hospitalised Female Adolescents with Eating Disorders. Int. J. Psychiatry Clin. Pract..

[73] Lewis Y.D., Mann T.G., Enoch-Levy A., Dubnov-Raz G., Gothelf D., Weizman A., Stein D. (2019). Obsessive-Compulsive Symptomatology in Female Adolescent Inpatients with Restrictive Compared with Binge-Purge Eating Disorders. Eur. Eat. Disord. Rev..

[74] Addolorato G., Ancona C., Capristo E., Graziosetto R., di Rienzo L., Maurizi M., Gasbarrini G. (1999). State and Trait Anxiety in Women Affected by Allergic and Vasomotor Rhinitis. J. Psychosom. Res..

[75] Hay P.J., Touyz S., Sud R. (2012). Treatment for Severe and Enduring Anorexia Nervosa: A Review. Aust. N. Z. J. Psychiatry.

[76] Fichter M.M., Leibl C., Krüger R., Rief W. (1997). Effects of Fluvoxamine on Depression, Anxiety, and Other Areas of General Psychopathology in Bulimia Nervosa. Pharmacopsychiatry.

[77] Claes L., Vandereycken W. (2007). Is There a Link between Traumatic Experiences and Self-Injurious Behaviors in Eating-Disordered Patients?. Eat. Disord..

[78] Bulik C.M., Thornton L., Pinheiro A.P., Plotnicov K., Klump K.L., Brandt H., Crawford S., Fichter M.M., Halmi K.A., Johnson C. (2008). Suicide Attempts in Anorexia Nervosa. Psychosom. Med..

[79] Forrest L.N., Smith A.R., Swanson S.A. (2017). Characteristics of Seeking Treatment among U.S. Adolescents with Eating Disorders. Int. J. Eat. Disord..

